# 1,2-Bis{[3,5-bis­(2,6-diisopropyl­phen­yl)phen­yl]imino}­acenaphthene toluene monosolvate

**DOI:** 10.1107/S1600536811031242

**Published:** 2011-08-11

**Authors:** Tracy L. Lohr, Warren E. Piers, Masood Parvez

**Affiliations:** a Department of Chemistry, The University of Calgary, 2500 University Drive NW, Calgary, Alberta, Canada T2N 1N4

## Abstract

In the title compound, C_72_H_80_N_2_·C_7_H_8_, the acenaphthene ring system is essentially planar, with a maximum deviation of 0.041 (3) Å. The benzene rings bonded to the the N atoms are essentially parallel, forming a dihedral angle of 0.80 (11)°, and these rings form dihedral angles of 87.49 (9) and 88.25 (10)° with the mean plane of the acenaphthene ring system. The methyl C atoms of three of the isopropyl groups are disordered of two sets of sites of equal occupancy.

## Related literature

For background to water splitting, see: Yang & Hall (2010 ▶); Kee *et al.* (2011 ▶); Blakemore *et al.* (2010 ▶). For related structures, see: El-Ayaan *et al.* (2003 ▶, 2004 ▶); Fedushkin *et al.* (2004 ▶); Coventry *et al.* (2004 ▶); Lohr *et al.* (2011 ▶).
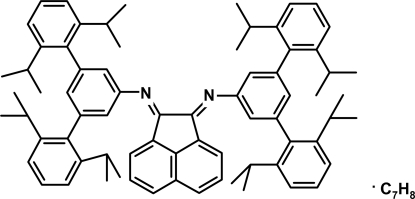


## Experimental

### 

#### Crystal data

C_72_H_80_N_2_·C_7_H_8_
*M* *_r_* = 1065.51
Triclinic, 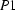

*a* = 10.4847 (2) Å
*b* = 10.8571 (3) Å
*c* = 14.8431 (3) Å
α = 77.447 (1)°
β = 82.283 (1)°
γ = 85.908 (1)°
*V* = 1632.76 (6) Å^3^
*Z* = 1
Cu *K*α radiation
μ = 0.46 mm^−1^
*T* = 100 K
0.20 × 0.20 × 0.20 mm

#### Data collection

Bruker APEXII CCD diffractometer
Absorption correction: multi-scan (*SADABS*; Bruker, 2004 ▶) *T*_min_ = 0.914, *T*_max_ = 0.914
25750 measured reflections
5747 independent reflections
5578 reflections with *I* > 2σ(*I*)
*R*_int_ = 0.036

#### Refinement

*R*[*F* ^2^ > 2σ(*F* ^2^)] = 0.059
*wR*(*F* ^2^) = 0.163
*S* = 1.05
5747 reflections
763 parameters
15 restraints
H-atom parameters constrained
Δρ_max_ = 0.47 e Å^−3^
Δρ_min_ = −0.26 e Å^−3^

### 

Data collection: *APEX2* (Bruker, 2004 ▶); cell refinement: *SAINT* (Bruker, 2004 ▶); data reduction: *SAINT*; program(s) used to solve structure: *SHELXS97* (Sheldrick, 2008 ▶); program(s) used to refine structure: *SHELXL97* (Sheldrick, 2008 ▶); molecular graphics: *ORTEP-3 for Windows* (Farrugia, 1997 ▶); software used to prepare material for publication: *SHELXL97* and *PLATON* (Spek, 2009 ▶).

## Supplementary Material

Crystal structure: contains datablock(s) global, I. DOI: 10.1107/S1600536811031242/lh5296sup1.cif

Structure factors: contains datablock(s) I. DOI: 10.1107/S1600536811031242/lh5296Isup2.hkl

Supplementary material file. DOI: 10.1107/S1600536811031242/lh5296Isup3.cml

Additional supplementary materials:  crystallographic information; 3D view; checkCIF report

## Figures and Tables

**Fig. 1.**
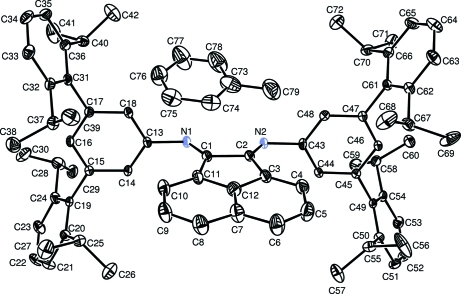
ORTEP drawing (Farrugia, 1997) of the title molecule with the displacement ellipsoids plotted at 30% probability level; H-atoms have been excluded for clarity. The disorder is not shown.

## supplementary crystallographic information

### Comment

To meet the ever-growing demand for green and carbon neutral energy, water splitting for the generation of hydrogen fuel represents an appealing strategy. To do their part, chemists have been looking at the steps towards organometallic mono-nuclear water splitting (Yang & Hall, 2010; Kee *et al.*, 2011; Blakemore *et al.*, 2010). Our group is currently exploring the use of platinum to mediate this reaction in an effort to understand the fundamental steps of O—H and O—O bond activation. This research is directed at synthesizing and studying plausible intermediates (Pt–OH and Pt–H species) in order to determine what role they play in the water activation process. The title compound was synthesized as a ligand to stabilize and isolate these highly reactive monomeric species for further mechanistic study.

In the title compound (Fig. 1.), the acenaphthene ring system (C1–C12) is essentially planar with the maximum deviation of C3 being 0.041 (3) Å. The benzene rings (C13–C18) and (C43–C48) bonded to N1 and N2, respectively, lie essentially parallel to each other with a dihedral angle between the two rings of 0.80 (11)°. The benzene rings (C13–C18) and (C43–C48) are oriented at approximately right angles [87.49 (9) and 88.25 (10)°, respectively] with respect to the mean-plane of the acenaphthene ring system. The dihedral angles between the benzene ring bonded to N1 and rings C19–C24 and C31–C36 are: 79.15 (12) and 76.89 (12)°, respectively. The corresponding angles between the benzene ring bonded to N2 and rings C49–C54 and C61–C66 are: 77.48 (12) and 79.83 (13)°, respectively. The molecular dimesions in the title compound agree very well with the corresponding molecular dimensions reported in a few closely related compounds (El-Ayaan *et al.*, 2003, 2004; Fedushkin *et al.*, 2004; Coventry *et al.*, 2004; Lohr *et al.*, 2011).

### Experimental

3,5-bis(2,6-Diisopropylphenyl)aniline (1.06 g, 2.56 mmol) and 1,2-acenaphthenequinone (0.186 g, 1.02 mmol) were dissolved in toluene (50 ml). To this orange solution were added 3 drops of formic acid and 4 A° mol. sieves, and the mixture was heated at 363 K for 14 hr. The mixture was cooled, filtered, and concentrated to yield an orange solid. MeOH (50 ml), toluene (2 ml), and 3 drops of formic acid were added, and the solution was stirred for 12 h. This was filtered and washed with cold MeOH (3 *x* 10 ml), and dried over an aspirator for 3 hr to yield a pale yellow solid (0.484 g, 49%). X-ray quality crystals were obtained by slow evaporation of a solution of the title compound in ethyl acetate:toluene (2:1).

### Refinement

although the H-atoms were visible in difference Fourier maps they were included in geometrically idealized positions with C—H distances = 0.95, 0.98 and 1.00 Å for aryl, methine and methyl type H-atoms, respectively. The H-atoms were assigned *U*_iso_ = 1.2 times *U*_eq_ of the aryl, methine C atoms and 1.5 times *U*_eq_ of the methyl C atoms. The methyl C-atoms of three isopropyl groups, C28–C30, C55–C57 and C67–C69 were disordered; EADP commands were used to model these groups with equal site occupancy factors. *PLATON* (Spek, 2009) suggested a pseudo center of symmetry indicating P -1 as the alternate space group which was not supported by the analysis of the structure. Since the compound contains no heavy atom an absolute configuration could not be determined; the Friedel pairs (2781) were merged.

### Crystal data

**Table:**

C_72_H_80_N_2_·C_7_H_8_	*Z* = 1
*M**_r_* = 1065.51	*F*(000) = 576
Triclinic, *P*1	*D*_x_ = 1.084 Mg m^−^^3^
Hall symbol: P 1	Cu *K*α radiation, λ = 1.54178 Å
*a* = 10.4847 (2) Å	Cell parameters from 16090 reflections
*b* = 10.8571 (3) Å	θ = 4.0–68.0°
*c* = 14.8431 (3) Å	µ = 0.46 mm^−^^1^
α = 77.447 (1)°	*T* = 100 K
β = 82.283 (1)°	Prism, yellow
γ = 85.908 (1)°	0.20 × 0.20 × 0.20 mm
*V* = 1632.76 (6) Å^3^	

### Data collection

**Table:**

Bruker APEXII CCD diffractometer	5747 independent reflections
Radiation source: fine-focus sealed tube	5578 reflections with *I* > 2σ(*I*)
graphite	*R*_int_ = 0.036
ω and φ scans	θ_max_ = 68.0°, θ_min_ = 4.2°
Absorption correction: multi-scan (*SADABS*; Bruker, 2004)	*h* = −12→12
*T*_min_ = 0.914, *T*_max_ = 0.914	*k* = −10→12
25750 measured reflections	*l* = −17→17

### Refinement

**Table:**

Refinement on *F*^2^	Primary atom site location: structure-invariant direct methods
Least-squares matrix: full	Secondary atom site location: difference Fourier map
*R*[*F*^2^ > 2σ(*F*^2^)] = 0.059	Hydrogen site location: inferred from neighbouring sites
*wR*(*F*^2^) = 0.163	H-atom parameters constrained
*S* = 1.05	*w* = 1/[σ^2^(*F*_o_^2^) + (0.1012*P*)^2^ + 0.8175*P*] where *P* = (*F*_o_^2^ + 2*F*_c_^2^)/3
5747 reflections	(Δ/σ)_max_ = 0.002
763 parameters	Δρ_max_ = 0.47 e Å^−^^3^
15 restraints	Δρ_min_ = −0.26 e Å^−^^3^

### Special details

**Table:**

Experimental. ^1^H NMR (CD_2_Cl_2_): δ = 1.09 (d, 24H, CH(C*H*_3_)_2_), 1.15 (d, 24H, CH(C*H*_3_)_2_), 2.99 (m, 8H, C*H*(CH_3_)_2_), 6.85 (d, 4H, Ar–H), 6.87 (d, 2H, Ar–H), 7.17 (d, 2H, Napth–H), 7.19 (d, 8H, Ar–H), 7.31 (t, 4H, Ar–H), 7.46 (t, 2H, Napth–H), 7.99 (d, 2H, Napth–H). ^13^C NMR (100 Mz, CD_2_Cl_2_): δ = 24.5 (CH(*C*H_3_)_2_), 24.6 (CH(*C*H_3_)_2_, 31.11 (*C*H(CH_3_)_2_, 117.33 (Ar–CH), 123.01 (Ar–CH), 124.58 (Ar–CH), 127.01 (Ar–C), 127.62 (Ar–CH), 127.86 (Ar–CH), 128.46 (Ar–CH), 129.45 (Ar–C), 129.70 (Ar–CH), 131.90 (Ar–C), 139.56 (Ar–C), 142.66 (Ar–C), 147.25 (Ar–C), 152.67 (Ar–C), 161.70 (N═*C*–Ar). ESIMS, Calcd for C_72_H_81_N_2_ ([M + H]+): 973.6394. Found: 973.6398. Anal. Calcd for C_72_H_80_N_2_: C, 88.84; H, 8.28; N, 2.88. Found: C, 81.50; H, 8.20; N, 2.20.
Geometry. All e.s.d.'s (except the e.s.d. in the dihedral angle between two l.s. planes) are estimated using the full covariance matrix. The cell e.s.d.'s are taken into account individually in the estimation of e.s.d.'s in distances, angles and torsion angles; correlations between e.s.d.'s in cell parameters are only used when they are defined by crystal symmetry. An approximate (isotropic) treatment of cell e.s.d.'s is used for estimating e.s.d.'s involving l.s. planes.
Refinement. Refinement of *F*^2^ against ALL reflections. The weighted *R*-factor *wR* and goodness of fit *S* are based on *F*^2^, conventional *R*-factors *R* are based on *F*, with *F* set to zero for negative *F*^2^. The threshold expression of *F*^2^ > σ(*F*^2^) is used only for calculating *R*-factors(gt) *etc*. and is not relevant to the choice of reflections for refinement. *R*-factors based on *F*^2^ are statistically about twice as large as those based on *F*, and *R*- factors based on ALL data will be even larger.

### Fractional atomic coordinates and isotropic or equivalent isotropic displacement parameters (Å^2^)

**Table:**

	*x*	*y*	*z*	*U*_iso_*/*U*_eq_	Occ. (<1)
N1	0.5848 (3)	0.5049 (3)	0.41908 (19)	0.0255 (7)	
N2	0.3922 (3)	0.4791 (3)	0.5725 (2)	0.0275 (7)	
C1	0.5565 (3)	0.3978 (3)	0.4673 (2)	0.0272 (7)	
C2	0.4495 (3)	0.3834 (4)	0.5487 (2)	0.0283 (7)	
C3	0.4445 (3)	0.2465 (4)	0.5901 (3)	0.0334 (8)	
C4	0.3738 (4)	0.1751 (4)	0.6658 (3)	0.0425 (10)	
H4	0.3051	0.2123	0.7009	0.051*	
C5	0.4068 (5)	0.0450 (5)	0.6895 (4)	0.0562 (13)	
H5	0.3602	−0.0039	0.7431	0.067*	
C6	0.4998 (5)	−0.0144 (5)	0.6411 (4)	0.0613 (14)	
H6	0.5160	−0.1029	0.6599	0.074*	
C7	0.5742 (5)	0.0557 (5)	0.5613 (4)	0.0509 (11)	
C8	0.6780 (5)	0.0089 (5)	0.5030 (4)	0.0571 (12)	
H8	0.7017	−0.0787	0.5150	0.069*	
C9	0.7429 (5)	0.0881 (5)	0.4308 (4)	0.0568 (13)	
H9	0.8115	0.0547	0.3933	0.068*	
C10	0.7108 (4)	0.2205 (4)	0.4097 (3)	0.0435 (10)	
H10	0.7571	0.2743	0.3585	0.052*	
C11	0.6123 (3)	0.2691 (4)	0.4642 (3)	0.0346 (8)	
C12	0.5430 (4)	0.1852 (5)	0.5392 (3)	0.0414 (10)	
C13	0.6853 (3)	0.5180 (3)	0.3435 (2)	0.0236 (7)	
C14	0.8093 (3)	0.5390 (4)	0.3577 (2)	0.0239 (7)	
H14	0.8275	0.5414	0.4183	0.029*	
C15	0.9078 (3)	0.5568 (3)	0.2829 (2)	0.0221 (7)	
C16	0.8778 (3)	0.5534 (3)	0.1944 (2)	0.0221 (7)	
H16	0.9439	0.5645	0.1432	0.027*	
C17	0.7537 (3)	0.5344 (3)	0.1800 (2)	0.0214 (7)	
C18	0.6570 (3)	0.5165 (3)	0.2552 (2)	0.0243 (7)	
H18	0.5715	0.5033	0.2459	0.029*	
C19	1.0421 (3)	0.5725 (3)	0.2995 (2)	0.0226 (7)	
C20	1.1157 (3)	0.4645 (4)	0.3365 (2)	0.0267 (8)	
C21	1.2384 (4)	0.4797 (4)	0.3567 (3)	0.0356 (9)	
H21	1.2885	0.4076	0.3828	0.043*	
C22	1.2887 (4)	0.5975 (5)	0.3395 (3)	0.0423 (10)	
H22	1.3724	0.6062	0.3545	0.051*	
C23	1.2183 (4)	0.7023 (4)	0.3008 (3)	0.0385 (9)	
H23	1.2547	0.7828	0.2880	0.046*	
C24	1.0944 (3)	0.6927 (4)	0.2798 (3)	0.0293 (8)	
C25	1.0650 (4)	0.3336 (4)	0.3546 (3)	0.0327 (9)	
H25	0.9757	0.3429	0.3367	0.039*	
C26	1.0559 (5)	0.2666 (5)	0.4560 (3)	0.0505 (12)	
H26A	1.1420	0.2557	0.4761	0.076*	
H26B	1.0198	0.1836	0.4635	0.076*	
H26C	0.9999	0.3171	0.4939	0.076*	
C27	1.1450 (6)	0.2530 (5)	0.2934 (4)	0.0580 (14)	
H27A	1.2335	0.2418	0.3092	0.087*	
H27B	1.1455	0.2953	0.2279	0.087*	
H27C	1.1073	0.1703	0.3037	0.087*	
C28	1.0181 (4)	0.8102 (4)	0.2379 (3)	0.0415 (11)	
H28	0.9297	0.7958	0.2251	0.050*	0.50
C29	1.0262 (10)	0.9142 (8)	0.2935 (6)	0.053 (2)	0.50
H29A	0.9965	0.8817	0.3594	0.080*	0.50
H29B	0.9716	0.9879	0.2694	0.080*	0.50
H29C	1.1156	0.9389	0.2869	0.080*	0.50
C30	1.1009 (11)	0.8927 (10)	0.1580 (6)	0.054 (2)	0.50
H30A	1.1641	0.9340	0.1831	0.081*	0.50
H30B	1.0457	0.9571	0.1228	0.081*	0.50
H30C	1.1459	0.8401	0.1168	0.081*	0.50
H28'	0.9421	0.7644	0.2303	0.050*	0.50
C29'	0.9466 (11)	0.8825 (10)	0.3037 (6)	0.053 (2)	0.50
H29D	1.0075	0.9143	0.3370	0.080*	0.50
H29E	0.8868	0.8274	0.3485	0.080*	0.50
H29F	0.8980	0.9538	0.2693	0.080*	0.50
C30'	1.0538 (14)	0.8613 (12)	0.1362 (4)	0.054 (2)	0.50
H30D	1.0045	0.9407	0.1170	0.081*	0.50
H30E	1.0342	0.7999	0.1009	0.081*	0.50
H30F	1.1461	0.8767	0.1240	0.081*	0.50
C31	0.7236 (3)	0.5294 (3)	0.0850 (2)	0.0230 (7)	
C32	0.7585 (3)	0.4198 (4)	0.0505 (2)	0.0266 (8)	
C33	0.7305 (4)	0.4178 (4)	−0.0387 (3)	0.0335 (9)	
H33	0.7554	0.3453	−0.0640	0.040*	
C34	0.6676 (4)	0.5186 (4)	−0.0903 (3)	0.0376 (10)	
H34	0.6482	0.5149	−0.1504	0.045*	
C35	0.6326 (4)	0.6252 (4)	−0.0548 (3)	0.0329 (9)	
H35	0.5886	0.6942	−0.0908	0.040*	
C36	0.6603 (3)	0.6336 (4)	0.0322 (2)	0.0261 (8)	
C37	0.8243 (4)	0.3047 (4)	0.1075 (3)	0.0314 (8)	
H37	0.8256	0.3216	0.1710	0.038*	
C38	0.9635 (4)	0.2839 (5)	0.0673 (4)	0.0460 (11)	
H38A	1.0029	0.2110	0.1070	0.069*	
H38B	1.0113	0.3592	0.0644	0.069*	
H38C	0.9659	0.2679	0.0046	0.069*	
C39	0.7491 (5)	0.1869 (5)	0.1190 (4)	0.0550 (13)	
H39A	0.7430	0.1694	0.0577	0.083*	
H39B	0.6623	0.2001	0.1503	0.083*	
H39C	0.7934	0.1152	0.1565	0.083*	
C40	0.6245 (4)	0.7549 (4)	0.0687 (3)	0.0299 (8)	
H40	0.6554	0.7428	0.1311	0.036*	
C41	0.6927 (6)	0.8679 (5)	0.0047 (4)	0.0628 (15)	
H41A	0.6635	0.8826	−0.0569	0.094*	
H41B	0.7861	0.8501	−0.0009	0.094*	
H41C	0.6721	0.9433	0.0310	0.094*	
C42	0.4819 (4)	0.7822 (6)	0.0812 (4)	0.0566 (14)	
H42A	0.4480	0.7883	0.0219	0.085*	
H42B	0.4638	0.8622	0.1017	0.085*	
H42C	0.4407	0.7139	0.1280	0.085*	
C43	0.2950 (3)	0.4617 (3)	0.6495 (2)	0.0246 (7)	
C44	0.3244 (3)	0.4675 (4)	0.7373 (2)	0.0243 (7)	
H44	0.4094	0.4844	0.7452	0.029*	
C45	0.2291 (3)	0.4485 (3)	0.8134 (2)	0.0226 (7)	
C46	0.1041 (3)	0.4258 (3)	0.8000 (2)	0.0226 (7)	
H46	0.0389	0.4119	0.8517	0.027*	
C47	0.0740 (3)	0.4233 (3)	0.7121 (2)	0.0229 (7)	
C48	0.1696 (3)	0.4409 (4)	0.6372 (2)	0.0256 (7)	
H48	0.1495	0.4388	0.5770	0.031*	
C49	0.2591 (3)	0.4535 (3)	0.9084 (2)	0.0217 (7)	
C50	0.3208 (3)	0.3485 (4)	0.9611 (2)	0.0274 (8)	
C51	0.3461 (4)	0.3562 (4)	1.0502 (3)	0.0325 (9)	
H51	0.3890	0.2870	1.0868	0.039*	
C52	0.3100 (4)	0.4624 (4)	1.0852 (3)	0.0348 (9)	
H52	0.3283	0.4658	1.1456	0.042*	
C53	0.2470 (4)	0.5648 (4)	1.0331 (3)	0.0303 (8)	
H53	0.2211	0.6372	1.0584	0.036*	
C54	0.2217 (3)	0.5617 (4)	0.9440 (2)	0.0252 (7)	
C55	0.3569 (3)	0.2292 (4)	0.9257 (3)	0.0336 (9)	
H55	0.3339	0.2577	0.8610	0.040*	0.50
C56	0.2621 (14)	0.1261 (14)	0.9622 (12)	0.070 (4)	0.50
H56A	0.2923	0.0513	0.9372	0.105*	0.50
H56B	0.1776	0.1558	0.9428	0.105*	0.50
H56C	0.2550	0.1044	1.0303	0.105*	0.50
C57	0.5003 (8)	0.204 (3)	0.9040 (14)	0.053 (4)	0.50
H57A	0.5437	0.2842	0.8877	0.080*	0.50
H57B	0.5164	0.1610	0.8517	0.080*	0.50
H57C	0.5335	0.1503	0.9587	0.080*	0.50
H55'	0.3361	0.2358	0.8610	0.040*	0.50
C56'	0.2964 (15)	0.1171 (14)	0.9940 (10)	0.070 (4)	0.50
H56D	0.3206	0.0398	0.9708	0.105*	0.50
H56E	0.2025	0.1300	1.0008	0.105*	0.50
H56F	0.3272	0.1094	1.0546	0.105*	0.50
C57'	0.4946 (9)	0.182 (3)	0.9374 (13)	0.053 (4)	0.50
H57D	0.5127	0.1042	0.9141	0.080*	0.50
H57E	0.5064	0.1656	1.0034	0.080*	0.50
H57F	0.5537	0.2466	0.9023	0.080*	0.50
C58	0.1569 (4)	0.6769 (4)	0.8866 (2)	0.0286 (8)	
H58	0.1601	0.6625	0.8220	0.034*	
C59	0.2272 (5)	0.7967 (4)	0.8805 (4)	0.0462 (11)	
H59A	0.1922	0.8654	0.8347	0.069*	
H59B	0.3192	0.7822	0.8614	0.069*	
H59C	0.2155	0.8197	0.9414	0.069*	
C60	0.0160 (4)	0.6931 (5)	0.9242 (3)	0.0442 (11)	
H60A	−0.0273	0.6144	0.9292	0.066*	
H60B	−0.0254	0.7616	0.8819	0.066*	
H60C	0.0099	0.7137	0.9858	0.066*	
C61	−0.0618 (3)	0.4027 (4)	0.6991 (2)	0.0253 (8)	
C62	−0.1084 (3)	0.2805 (4)	0.7220 (3)	0.0300 (8)	
C63	−0.2357 (4)	0.2660 (4)	0.7109 (3)	0.0426 (10)	
H63	−0.2697	0.1843	0.7266	0.051*	
C64	−0.3133 (4)	0.3698 (5)	0.6773 (4)	0.0476 (11)	
H64	−0.4002	0.3585	0.6703	0.057*	
C65	−0.2670 (4)	0.4876 (5)	0.6543 (3)	0.0406 (10)	
H65	−0.3220	0.5572	0.6307	0.049*	
C66	−0.1400 (4)	0.5088 (4)	0.6646 (2)	0.0288 (8)	
C67	−0.0255 (4)	0.1643 (4)	0.7582 (3)	0.0371 (10)	
H67	0.0539	0.1943	0.7758	0.045*	0.50
C68	0.017 (2)	0.082 (3)	0.689 (2)	0.088 (6)	0.50
H68A	0.0577	0.1335	0.6310	0.132*	0.50
H68B	−0.0579	0.0424	0.6760	0.132*	0.50
H68C	0.0792	0.0160	0.7144	0.132*	0.50
C69	−0.095 (3)	0.086 (3)	0.8456 (7)	0.064 (5)	0.50
H69A	−0.1207	0.1388	0.8914	0.096*	0.50
H69B	−0.0384	0.0155	0.8716	0.096*	0.50
H69C	−0.1726	0.0528	0.8304	0.096*	0.50
H67'	0.0658	0.1899	0.7504	0.045*	0.50
C68'	−0.032 (2)	0.066 (3)	0.700 (2)	0.088 (6)	0.50
H68D	−0.0124	0.1056	0.6339	0.132*	0.50
H68E	−0.1187	0.0337	0.7113	0.132*	0.50
H68F	0.0310	−0.0031	0.7170	0.132*	0.50
C69'	−0.062 (3)	0.105 (3)	0.8597 (6)	0.064 (5)	0.50
H69D	−0.0043	0.0315	0.8780	0.096*	0.50
H69E	−0.1511	0.0785	0.8690	0.096*	0.50
H69F	−0.0546	0.1670	0.8977	0.096*	0.50
C70	−0.0927 (4)	0.6419 (4)	0.6433 (3)	0.0331 (9)	
H70	−0.0050	0.6365	0.6637	0.040*	
C71	−0.1775 (5)	0.7279 (4)	0.6971 (3)	0.0483 (11)	
H71A	−0.2648	0.7347	0.6792	0.072*	
H71B	−0.1418	0.8119	0.6829	0.072*	
H71C	−0.1807	0.6924	0.7640	0.072*	
C72	−0.0783 (5)	0.6970 (5)	0.5388 (3)	0.0485 (11)	
H72A	−0.0156	0.6446	0.5069	0.073*	
H72B	−0.0483	0.7832	0.5269	0.073*	
H72C	−0.1618	0.6987	0.5157	0.073*	
C73	0.3643 (7)	0.8745 (7)	0.4607 (6)	0.0812 (19)	
C74	0.4569 (6)	0.7920 (5)	0.4416 (5)	0.0625 (15)	
H74	0.4641	0.7108	0.4810	0.075*	
C75	0.5507 (7)	0.8303 (6)	0.3557 (6)	0.0793 (18)	
H75	0.6181	0.7729	0.3394	0.095*	
C76	0.5388 (7)	0.9438 (6)	0.3032 (5)	0.0791 (19)	
H76	0.5972	0.9693	0.2486	0.095*	
C77	0.4397 (8)	1.0260 (8)	0.3288 (8)	0.115 (3)	
H77	0.4318	1.1085	0.2915	0.139*	
C78	0.3510 (7)	0.9902 (7)	0.4084 (7)	0.102 (3)	
H78	0.2831	1.0471	0.4247	0.122*	
C79	0.2648 (7)	0.8277 (9)	0.5506 (7)	0.103 (3)	
H79A	0.1996	0.7782	0.5348	0.154*	
H79B	0.2227	0.9010	0.5727	0.154*	
H79C	0.3108	0.7751	0.5995	0.154*	

### Atomic displacement parameters (Å^2^)

**Table:**

	*U*^11^	*U*^22^	*U*^33^	*U*^12^	*U*^13^	*U*^23^
N1	0.0187 (14)	0.0383 (19)	0.0192 (14)	−0.0044 (12)	0.0031 (11)	−0.0075 (13)
N2	0.0176 (14)	0.043 (2)	0.0189 (14)	−0.0021 (13)	0.0023 (11)	−0.0039 (13)
C1	0.0200 (15)	0.040 (2)	0.0228 (15)	−0.0041 (13)	−0.0009 (12)	−0.0084 (14)
C2	0.0183 (15)	0.043 (2)	0.0233 (16)	−0.0032 (14)	−0.0013 (12)	−0.0070 (14)
C3	0.0231 (17)	0.044 (2)	0.0333 (19)	−0.0047 (15)	−0.0003 (14)	−0.0091 (16)
C4	0.034 (2)	0.042 (2)	0.047 (2)	−0.0067 (17)	0.0013 (17)	−0.0034 (18)
C5	0.060 (3)	0.046 (3)	0.053 (3)	−0.008 (2)	0.014 (2)	0.001 (2)
C6	0.062 (3)	0.040 (3)	0.074 (3)	−0.012 (2)	0.017 (3)	−0.006 (2)
C7	0.045 (2)	0.042 (3)	0.062 (3)	−0.0057 (19)	0.009 (2)	−0.013 (2)
C8	0.053 (3)	0.042 (3)	0.074 (3)	0.003 (2)	0.010 (2)	−0.018 (2)
C9	0.047 (3)	0.057 (3)	0.065 (3)	0.001 (2)	0.016 (2)	−0.024 (2)
C10	0.036 (2)	0.052 (3)	0.043 (2)	−0.0009 (18)	0.0088 (17)	−0.0205 (19)
C11	0.0253 (17)	0.047 (2)	0.0324 (19)	−0.0056 (15)	0.0029 (14)	−0.0137 (16)
C12	0.039 (2)	0.042 (2)	0.044 (2)	−0.0069 (18)	−0.0040 (18)	−0.0081 (19)
C13	0.0157 (15)	0.0288 (19)	0.0226 (17)	−0.0012 (13)	0.0031 (13)	−0.0009 (14)
C14	0.0205 (16)	0.033 (2)	0.0163 (16)	−0.0013 (14)	0.0026 (12)	−0.0051 (13)
C15	0.0181 (16)	0.0261 (18)	0.0205 (16)	−0.0001 (13)	0.0002 (13)	−0.0033 (13)
C16	0.0192 (16)	0.0261 (18)	0.0188 (16)	−0.0013 (13)	0.0049 (12)	−0.0042 (13)
C17	0.0208 (16)	0.0200 (17)	0.0210 (17)	0.0002 (13)	0.0008 (13)	−0.0018 (13)
C18	0.0154 (15)	0.033 (2)	0.0232 (17)	−0.0048 (13)	0.0019 (13)	−0.0039 (14)
C19	0.0181 (16)	0.033 (2)	0.0151 (15)	0.0002 (13)	0.0020 (12)	−0.0040 (13)
C20	0.0229 (17)	0.032 (2)	0.0263 (18)	0.0029 (14)	−0.0032 (13)	−0.0085 (15)
C21	0.0243 (18)	0.044 (2)	0.039 (2)	0.0062 (16)	−0.0108 (15)	−0.0060 (17)
C22	0.0173 (17)	0.053 (3)	0.059 (3)	−0.0006 (17)	−0.0102 (16)	−0.013 (2)
C23	0.0224 (18)	0.044 (2)	0.049 (2)	−0.0075 (16)	−0.0001 (16)	−0.0100 (19)
C24	0.0209 (17)	0.032 (2)	0.0319 (19)	−0.0047 (15)	0.0016 (14)	−0.0027 (16)
C25	0.034 (2)	0.033 (2)	0.0303 (19)	0.0014 (16)	−0.0064 (16)	−0.0028 (16)
C26	0.068 (3)	0.051 (3)	0.029 (2)	−0.013 (2)	−0.003 (2)	−0.0002 (19)
C27	0.079 (4)	0.040 (3)	0.054 (3)	−0.001 (2)	0.004 (3)	−0.015 (2)
C28	0.032 (2)	0.032 (2)	0.054 (3)	−0.0044 (17)	−0.0039 (19)	0.0061 (19)
C29	0.052 (4)	0.063 (5)	0.032 (3)	0.026 (4)	0.008 (4)	0.000 (3)
C30	0.085 (7)	0.046 (5)	0.029 (4)	0.020 (4)	−0.010 (4)	−0.007 (3)
C28'	0.032 (2)	0.032 (2)	0.054 (3)	−0.0044 (17)	−0.0039 (19)	0.0061 (19)
C29'	0.052 (4)	0.063 (5)	0.032 (3)	0.026 (4)	0.008 (4)	0.000 (3)
C30'	0.085 (7)	0.046 (5)	0.029 (4)	0.020 (4)	−0.010 (4)	−0.007 (3)
C31	0.0175 (15)	0.0286 (19)	0.0214 (17)	−0.0066 (13)	0.0033 (13)	−0.0037 (14)
C32	0.0265 (18)	0.029 (2)	0.0227 (17)	−0.0062 (14)	0.0019 (14)	−0.0045 (14)
C33	0.040 (2)	0.037 (2)	0.0255 (19)	−0.0074 (17)	0.0000 (16)	−0.0122 (16)
C34	0.047 (2)	0.048 (3)	0.0196 (18)	−0.011 (2)	−0.0058 (16)	−0.0056 (17)
C35	0.034 (2)	0.036 (2)	0.0266 (19)	−0.0047 (16)	−0.0059 (15)	0.0013 (16)
C36	0.0236 (17)	0.031 (2)	0.0238 (17)	−0.0060 (14)	−0.0026 (13)	−0.0046 (15)
C37	0.034 (2)	0.031 (2)	0.0289 (19)	−0.0010 (16)	0.0007 (16)	−0.0069 (16)
C38	0.041 (2)	0.040 (3)	0.050 (3)	0.0070 (19)	0.0012 (19)	−0.001 (2)
C39	0.051 (3)	0.035 (3)	0.070 (3)	−0.008 (2)	−0.009 (2)	0.013 (2)
C40	0.0284 (18)	0.029 (2)	0.033 (2)	0.0037 (15)	−0.0070 (15)	−0.0061 (16)
C41	0.057 (3)	0.037 (3)	0.086 (4)	−0.012 (2)	0.021 (3)	−0.012 (2)
C42	0.033 (2)	0.061 (3)	0.084 (4)	0.000 (2)	−0.004 (2)	−0.036 (3)
C43	0.0208 (17)	0.031 (2)	0.0193 (17)	−0.0008 (14)	0.0062 (13)	−0.0044 (14)
C44	0.0151 (15)	0.034 (2)	0.0222 (17)	−0.0023 (13)	0.0004 (13)	−0.0049 (14)
C45	0.0201 (16)	0.0290 (19)	0.0177 (16)	−0.0017 (13)	−0.0015 (13)	−0.0029 (13)
C46	0.0164 (15)	0.0270 (18)	0.0212 (16)	−0.0020 (13)	0.0015 (12)	0.0001 (13)
C47	0.0182 (16)	0.0267 (18)	0.0216 (16)	−0.0017 (13)	0.0004 (13)	−0.0021 (13)
C48	0.0223 (17)	0.034 (2)	0.0190 (16)	−0.0012 (14)	−0.0025 (13)	−0.0032 (14)
C49	0.0166 (15)	0.0297 (19)	0.0167 (16)	−0.0064 (13)	0.0003 (12)	−0.0003 (13)
C50	0.0231 (17)	0.033 (2)	0.0238 (17)	−0.0022 (15)	−0.0034 (14)	0.0001 (15)
C51	0.034 (2)	0.038 (2)	0.0246 (19)	−0.0042 (17)	−0.0101 (15)	0.0010 (16)
C52	0.042 (2)	0.042 (2)	0.0221 (18)	−0.0076 (18)	−0.0092 (16)	−0.0042 (16)
C53	0.035 (2)	0.031 (2)	0.0256 (18)	−0.0066 (15)	−0.0014 (15)	−0.0070 (15)
C54	0.0240 (17)	0.0291 (19)	0.0198 (16)	−0.0058 (14)	0.0027 (13)	−0.0009 (14)
C55	0.037 (2)	0.034 (2)	0.0307 (19)	0.0034 (17)	−0.0126 (16)	−0.0056 (16)
C56	0.028 (7)	0.058 (4)	0.140 (12)	−0.021 (5)	0.017 (6)	−0.067 (6)
C57	0.030 (3)	0.053 (9)	0.077 (12)	−0.001 (3)	0.013 (4)	−0.026 (9)
C55'	0.037 (2)	0.034 (2)	0.0307 (19)	0.0034 (17)	−0.0126 (16)	−0.0056 (16)
C56'	0.028 (7)	0.058 (4)	0.140 (12)	−0.021 (5)	0.017 (6)	−0.067 (6)
C57'	0.030 (3)	0.053 (9)	0.077 (12)	−0.001 (3)	0.013 (4)	−0.026 (9)
C58	0.0306 (19)	0.029 (2)	0.0231 (17)	0.0001 (15)	−0.0002 (15)	−0.0024 (15)
C59	0.041 (2)	0.034 (2)	0.057 (3)	−0.0089 (18)	−0.008 (2)	0.007 (2)
C60	0.028 (2)	0.049 (3)	0.048 (3)	−0.0014 (18)	0.0006 (18)	0.003 (2)
C61	0.0172 (16)	0.038 (2)	0.0207 (16)	−0.0012 (14)	0.0004 (13)	−0.0076 (15)
C62	0.0211 (17)	0.035 (2)	0.0321 (19)	−0.0029 (15)	−0.0012 (14)	−0.0037 (16)
C63	0.027 (2)	0.040 (2)	0.062 (3)	−0.0086 (17)	−0.0046 (19)	−0.010 (2)
C64	0.0243 (19)	0.057 (3)	0.067 (3)	−0.0013 (18)	−0.0115 (19)	−0.022 (2)
C65	0.029 (2)	0.051 (3)	0.043 (2)	0.0096 (18)	−0.0107 (17)	−0.0130 (19)
C66	0.0268 (18)	0.038 (2)	0.0201 (17)	0.0009 (15)	−0.0015 (13)	−0.0050 (15)
C67	0.0251 (18)	0.032 (2)	0.052 (3)	−0.0041 (16)	−0.0068 (17)	−0.0005 (18)
C68	0.109 (17)	0.096 (8)	0.039 (7)	0.083 (10)	0.009 (10)	−0.012 (7)
C69	0.094 (14)	0.075 (9)	0.022 (3)	0.039 (6)	−0.014 (5)	−0.017 (5)
C67'	0.0251 (18)	0.032 (2)	0.052 (3)	−0.0041 (16)	−0.0068 (17)	−0.0005 (18)
C68'	0.109 (17)	0.096 (8)	0.039 (7)	0.083 (10)	0.009 (10)	−0.012 (7)
C69'	0.094 (14)	0.075 (9)	0.022 (3)	0.039 (6)	−0.014 (5)	−0.017 (5)
C70	0.037 (2)	0.032 (2)	0.0291 (19)	0.0067 (16)	−0.0093 (16)	−0.0039 (15)
C71	0.062 (3)	0.037 (3)	0.043 (2)	−0.001 (2)	0.005 (2)	−0.0098 (19)
C72	0.061 (3)	0.045 (3)	0.037 (2)	−0.003 (2)	−0.005 (2)	−0.0014 (19)
C73	0.070 (4)	0.065 (4)	0.117 (6)	0.009 (3)	−0.027 (4)	−0.032 (4)
C74	0.072 (4)	0.040 (3)	0.080 (4)	−0.005 (2)	−0.025 (3)	−0.012 (3)
C75	0.074 (4)	0.056 (4)	0.110 (5)	−0.008 (3)	−0.023 (4)	−0.015 (3)
C76	0.082 (4)	0.056 (4)	0.088 (5)	−0.008 (3)	0.007 (4)	−0.001 (3)
C77	0.081 (5)	0.068 (5)	0.176 (10)	−0.005 (4)	0.019 (5)	0.001 (5)
C78	0.070 (4)	0.067 (4)	0.155 (8)	0.011 (3)	0.003 (5)	−0.007 (5)
C79	0.066 (4)	0.129 (7)	0.116 (6)	−0.026 (5)	−0.002 (4)	−0.030 (6)

### Geometric parameters (Å, °)

**Table:**

N1—C1	1.258 (5)	C42—H42C	0.9800
N1—C13	1.422 (4)	C43—C48	1.392 (5)
N2—C2	1.256 (5)	C43—C44	1.393 (5)
N2—C43	1.415 (4)	C44—C45	1.394 (5)
C1—C11	1.483 (5)	C44—H44	0.9500
C1—C2	1.524 (4)	C45—C46	1.398 (5)
C2—C3	1.482 (6)	C45—C49	1.499 (5)
C3—C4	1.375 (6)	C46—C47	1.388 (5)
C3—C12	1.412 (6)	C46—H46	0.9500
C4—C5	1.409 (7)	C47—C48	1.383 (5)
C4—H4	0.9500	C47—C61	1.501 (5)
C5—C6	1.344 (8)	C48—H48	0.9500
C5—H5	0.9500	C49—C54	1.401 (5)
C6—C7	1.427 (7)	C49—C50	1.405 (5)
C6—H6	0.9500	C50—C51	1.404 (5)
C7—C12	1.398 (7)	C50—C55	1.506 (6)
C7—C8	1.432 (7)	C51—C52	1.374 (6)
C8—C9	1.355 (7)	C51—H51	0.9500
C8—H8	0.9500	C52—C53	1.388 (6)
C9—C10	1.429 (7)	C52—H52	0.9500
C9—H9	0.9500	C53—C54	1.392 (5)
C10—C11	1.372 (5)	C53—H53	0.9500
C10—H10	0.9500	C54—C58	1.524 (5)
C11—C12	1.426 (6)	C55—C56	1.511 (6)
C13—C18	1.386 (5)	C55—C57	1.513 (6)
C13—C14	1.387 (5)	C55—H55	1.0000
C14—C15	1.402 (5)	C56—H56A	0.9800
C14—H14	0.9500	C56—H56B	0.9800
C15—C16	1.399 (5)	C56—H56C	0.9800
C15—C19	1.490 (5)	C57—H57A	0.9800
C16—C17	1.383 (5)	C57—H57B	0.9800
C16—H16	0.9500	C57—H57C	0.9800
C17—C18	1.393 (5)	C56'—H56D	0.9800
C17—C31	1.499 (5)	C56'—H56E	0.9800
C18—H18	0.9500	C56'—H56F	0.9800
C19—C20	1.406 (5)	C57'—H57D	0.9800
C19—C24	1.408 (5)	C57'—H57E	0.9800
C20—C21	1.388 (5)	C57'—H57F	0.9800
C20—C25	1.510 (6)	C58—C60	1.519 (5)
C21—C22	1.378 (6)	C58—C59	1.520 (6)
C21—H21	0.9500	C58—H58	1.0000
C22—C23	1.371 (6)	C59—H59A	0.9800
C22—H22	0.9500	C59—H59B	0.9800
C23—C24	1.391 (5)	C59—H59C	0.9800
C23—H23	0.9500	C60—H60A	0.9800
C24—C28	1.516 (5)	C60—H60B	0.9800
C25—C26	1.516 (6)	C60—H60C	0.9800
C25—C27	1.527 (6)	C61—C62	1.403 (6)
C25—H25	1.0000	C61—C66	1.408 (5)
C26—H26A	0.9800	C62—C63	1.390 (5)
C26—H26B	0.9800	C62—C67	1.520 (5)
C26—H26C	0.9800	C63—C64	1.383 (7)
C27—H27A	0.9800	C63—H63	0.9500
C27—H27B	0.9800	C64—C65	1.357 (7)
C27—H27C	0.9800	C64—H64	0.9500
C28—C30	1.529 (6)	C65—C66	1.401 (6)
C28—C29	1.550 (6)	C65—H65	0.9500
C28—H28	1.0000	C66—C70	1.515 (6)
C29—H29A	0.9800	C67—C68	1.511 (6)
C29—H29B	0.9800	C67—C69	1.516 (6)
C29—H29C	0.9800	C67—H67	1.0000
C30—H30A	0.9800	C68—H68A	0.9800
C30—H30B	0.9800	C68—H68B	0.9800
C30—H30C	0.9800	C68—H68C	0.9800
C29'—H29D	0.9800	C69—H69A	0.9800
C29'—H29E	0.9800	C69—H69B	0.9800
C29'—H29F	0.9800	C69—H69C	0.9800
C30'—H30D	0.9800	C68'—H68D	0.9800
C30'—H30E	0.9800	C68'—H68E	0.9800
C30'—H30F	0.9800	C68'—H68F	0.9800
C31—C32	1.402 (5)	C69'—H69D	0.9800
C31—C36	1.408 (5)	C69'—H69E	0.9800
C32—C33	1.398 (5)	C69'—H69F	0.9800
C32—C37	1.526 (5)	C70—C71	1.522 (6)
C33—C34	1.374 (6)	C70—C72	1.527 (6)
C33—H33	0.9500	C70—H70	1.0000
C34—C35	1.380 (6)	C71—H71A	0.9800
C34—H34	0.9500	C71—H71B	0.9800
C35—C36	1.385 (5)	C71—H71C	0.9800
C35—H35	0.9500	C72—H72A	0.9800
C36—C40	1.535 (5)	C72—H72B	0.9800
C37—C39	1.516 (6)	C72—H72C	0.9800
C37—C38	1.522 (6)	C73—C74	1.319 (9)
C37—H37	1.0000	C73—C78	1.335 (10)
C38—H38A	0.9800	C73—C79	1.591 (12)
C38—H38B	0.9800	C74—C75	1.503 (10)
C38—H38C	0.9800	C74—H74	0.9500
C39—H39A	0.9800	C75—C76	1.315 (9)
C39—H39B	0.9800	C75—H75	0.9500
C39—H39C	0.9800	C76—C77	1.389 (11)
C40—C42	1.498 (6)	C76—H76	0.9500
C40—C41	1.535 (6)	C77—C78	1.403 (12)
C40—H40	1.0000	C77—H77	0.9500
C41—H41A	0.9800	C78—H78	0.9500
C41—H41B	0.9800	C79—H79A	0.9800
C41—H41C	0.9800	C79—H79B	0.9800
C42—H42A	0.9800	C79—H79C	0.9800
C42—H42B	0.9800		
			
C1—N1—C13	121.0 (3)	C44—C43—N2	120.0 (3)
C2—N2—C43	118.7 (3)	C43—C44—C45	120.0 (3)
N1—C1—C11	132.1 (3)	C43—C44—H44	120.0
N1—C1—C2	121.1 (3)	C45—C44—H44	120.0
C11—C1—C2	106.8 (3)	C44—C45—C46	119.1 (3)
N2—C2—C3	132.8 (3)	C44—C45—C49	120.7 (3)
N2—C2—C1	120.5 (3)	C46—C45—C49	120.1 (3)
C3—C2—C1	106.5 (3)	C47—C46—C45	120.9 (3)
C4—C3—C12	118.5 (4)	C47—C46—H46	119.6
C4—C3—C2	134.5 (4)	C45—C46—H46	119.6
C12—C3—C2	106.9 (3)	C48—C47—C46	119.5 (3)
C3—C4—C5	117.7 (4)	C48—C47—C61	120.6 (3)
C3—C4—H4	121.1	C46—C47—C61	119.8 (3)
C5—C4—H4	121.1	C47—C48—C43	120.3 (3)
C6—C5—C4	124.2 (5)	C47—C48—H48	119.9
C6—C5—H5	117.9	C43—C48—H48	119.9
C4—C5—H5	117.9	C54—C49—C50	121.2 (3)
C5—C6—C7	119.9 (5)	C54—C49—C45	119.3 (3)
C5—C6—H6	120.1	C50—C49—C45	119.5 (3)
C7—C6—H6	120.1	C51—C50—C49	117.9 (4)
C12—C7—C6	115.7 (4)	C51—C50—C55	120.1 (3)
C12—C7—C8	116.8 (4)	C49—C50—C55	122.1 (3)
C6—C7—C8	127.4 (5)	C52—C51—C50	121.1 (4)
C9—C8—C7	120.8 (5)	C52—C51—H51	119.5
C9—C8—H8	119.6	C50—C51—H51	119.5
C7—C8—H8	119.6	C51—C52—C53	120.6 (4)
C8—C9—C10	121.8 (4)	C51—C52—H52	119.7
C8—C9—H9	119.1	C53—C52—H52	119.7
C10—C9—H9	119.1	C52—C53—C54	120.2 (4)
C11—C10—C9	119.2 (4)	C52—C53—H53	119.9
C11—C10—H10	120.4	C54—C53—H53	119.9
C9—C10—H10	120.4	C53—C54—C49	119.1 (3)
C10—C11—C12	118.9 (4)	C53—C54—C58	119.4 (4)
C10—C11—C1	134.8 (4)	C49—C54—C58	121.5 (3)
C12—C11—C1	106.3 (3)	C50—C55—C56	113.9 (9)
C7—C12—C3	124.0 (4)	C50—C55—C57	114.5 (13)
C7—C12—C11	122.5 (4)	C56—C55—C57	123.6 (14)
C3—C12—C11	113.5 (4)	C50—C55—H55	99.5
C18—C13—C14	120.5 (3)	C56—C55—H55	99.5
C18—C13—N1	119.6 (3)	C57—C55—H55	99.5
C14—C13—N1	119.8 (3)	C55—C56—H56A	109.5
C13—C14—C15	120.1 (3)	C55—C56—H56B	109.5
C13—C14—H14	119.9	H56A—C56—H56B	109.5
C15—C14—H14	119.9	C55—C56—H56C	109.5
C16—C15—C14	118.6 (3)	H56A—C56—H56C	109.5
C16—C15—C19	121.5 (3)	H56B—C56—H56C	109.5
C14—C15—C19	119.8 (3)	C55—C57—H57A	109.5
C17—C16—C15	121.2 (3)	C55—C57—H57B	109.5
C17—C16—H16	119.4	H57A—C57—H57B	109.5
C15—C16—H16	119.4	C55—C57—H57C	109.5
C16—C17—C18	119.5 (3)	H57A—C57—H57C	109.5
C16—C17—C31	120.5 (3)	H57B—C57—H57C	109.5
C18—C17—C31	120.0 (3)	H56D—C56'—H56E	109.5
C13—C18—C17	120.1 (3)	H56D—C56'—H56F	109.5
C13—C18—H18	120.0	H56E—C56'—H56F	109.5
C17—C18—H18	120.0	H57D—C57'—H57E	109.5
C20—C19—C24	120.3 (3)	H57D—C57'—H57F	109.5
C20—C19—C15	118.5 (3)	H57E—C57'—H57F	109.5
C24—C19—C15	121.1 (3)	C60—C58—C59	110.5 (4)
C21—C20—C19	118.7 (4)	C60—C58—C54	111.4 (3)
C21—C20—C25	119.5 (3)	C59—C58—C54	111.9 (3)
C19—C20—C25	121.9 (3)	C60—C58—H58	107.6
C22—C21—C20	121.1 (4)	C59—C58—H58	107.6
C22—C21—H21	119.5	C54—C58—H58	107.6
C20—C21—H21	119.5	C58—C59—H59A	109.5
C23—C22—C21	120.2 (4)	C58—C59—H59B	109.5
C23—C22—H22	119.9	H59A—C59—H59B	109.5
C21—C22—H22	119.9	C58—C59—H59C	109.5
C22—C23—C24	121.2 (4)	H59A—C59—H59C	109.5
C22—C23—H23	119.4	H59B—C59—H59C	109.5
C24—C23—H23	119.4	C58—C60—H60A	109.5
C23—C24—C19	118.5 (4)	C58—C60—H60B	109.5
C23—C24—C28	120.1 (4)	H60A—C60—H60B	109.5
C19—C24—C28	121.3 (3)	C58—C60—H60C	109.5
C20—C25—C26	113.4 (4)	H60A—C60—H60C	109.5
C20—C25—C27	111.0 (4)	H60B—C60—H60C	109.5
C26—C25—C27	110.5 (4)	C62—C61—C66	121.6 (3)
C20—C25—H25	107.2	C62—C61—C47	120.1 (3)
C26—C25—H25	107.2	C66—C61—C47	118.3 (3)
C27—C25—H25	107.2	C63—C62—C61	118.2 (4)
C25—C26—H26A	109.5	C63—C62—C67	119.2 (4)
C25—C26—H26B	109.5	C61—C62—C67	122.6 (3)
H26A—C26—H26B	109.5	C64—C63—C62	120.5 (4)
C25—C26—H26C	109.5	C64—C63—H63	119.7
H26A—C26—H26C	109.5	C62—C63—H63	119.7
H26B—C26—H26C	109.5	C65—C64—C63	120.9 (4)
C25—C27—H27A	109.5	C65—C64—H64	119.6
C25—C27—H27B	109.5	C63—C64—H64	119.6
H27A—C27—H27B	109.5	C64—C65—C66	121.4 (4)
C25—C27—H27C	109.5	C64—C65—H65	119.3
H27A—C27—H27C	109.5	C66—C65—H65	119.3
H27B—C27—H27C	109.5	C65—C66—C61	117.3 (4)
C24—C28—C30	110.7 (6)	C65—C66—C70	120.4 (4)
C24—C28—C29	109.5 (5)	C61—C66—C70	122.2 (3)
C30—C28—C29	86.7 (6)	C68—C67—C69	109.5 (18)
C24—C28—H28	115.5	C68—C67—C62	114.6 (14)
C30—C28—H28	115.5	C69—C67—C62	110.0 (15)
C29—C28—H28	115.5	C68—C67—H67	107.5
H29D—C29'—H29E	109.5	C69—C67—H67	107.5
H29D—C29'—H29F	109.5	C62—C67—H67	107.5
H29E—C29'—H29F	109.5	C67—C68—H68A	109.5
H30D—C30'—H30E	109.5	C67—C68—H68B	109.5
H30D—C30'—H30F	109.5	H68A—C68—H68B	109.5
H30E—C30'—H30F	109.5	C67—C68—H68C	109.5
C32—C31—C36	121.0 (3)	H68A—C68—H68C	109.5
C32—C31—C17	119.1 (3)	H68B—C68—H68C	109.5
C36—C31—C17	119.9 (3)	C67—C69—H69A	109.5
C33—C32—C31	118.0 (3)	C67—C69—H69B	109.5
C33—C32—C37	120.1 (4)	H69A—C69—H69B	109.5
C31—C32—C37	121.8 (3)	C67—C69—H69C	109.5
C34—C33—C32	121.3 (4)	H69A—C69—H69C	109.5
C34—C33—H33	119.3	H69B—C69—H69C	109.5
C32—C33—H33	119.3	H68D—C68'—H68E	109.5
C33—C34—C35	119.9 (4)	H68D—C68'—H68F	109.5
C33—C34—H34	120.0	H68E—C68'—H68F	109.5
C35—C34—H34	120.0	H69D—C69'—H69E	109.5
C34—C35—C36	121.3 (4)	H69D—C69'—H69F	109.5
C34—C35—H35	119.3	H69E—C69'—H69F	109.5
C36—C35—H35	119.3	C66—C70—C71	112.3 (4)
C35—C36—C31	118.4 (3)	C66—C70—C72	111.0 (4)
C35—C36—C40	120.4 (3)	C71—C70—C72	111.5 (4)
C31—C36—C40	121.3 (3)	C66—C70—H70	107.3
C39—C37—C38	111.1 (4)	C71—C70—H70	107.3
C39—C37—C32	111.6 (3)	C72—C70—H70	107.3
C38—C37—C32	112.1 (3)	C70—C71—H71A	109.5
C39—C37—H37	107.2	C70—C71—H71B	109.5
C38—C37—H37	107.2	H71A—C71—H71B	109.5
C32—C37—H37	107.2	C70—C71—H71C	109.5
C37—C38—H38A	109.5	H71A—C71—H71C	109.5
C37—C38—H38B	109.5	H71B—C71—H71C	109.5
H38A—C38—H38B	109.5	C70—C72—H72A	109.5
C37—C38—H38C	109.5	C70—C72—H72B	109.5
H38A—C38—H38C	109.5	H72A—C72—H72B	109.5
H38B—C38—H38C	109.5	C70—C72—H72C	109.5
C37—C39—H39A	109.5	H72A—C72—H72C	109.5
C37—C39—H39B	109.5	H72B—C72—H72C	109.5
H39A—C39—H39B	109.5	C74—C73—C78	123.3 (8)
C37—C39—H39C	109.5	C74—C73—C79	116.0 (7)
H39A—C39—H39C	109.5	C78—C73—C79	120.7 (7)
H39B—C39—H39C	109.5	C73—C74—C75	118.0 (6)
C42—C40—C36	112.3 (3)	C73—C74—H74	121.0
C42—C40—C41	110.4 (4)	C75—C74—H74	121.0
C36—C40—C41	110.8 (3)	C76—C75—C74	119.5 (7)
C42—C40—H40	107.7	C76—C75—H75	120.2
C36—C40—H40	107.7	C74—C75—H75	120.2
C41—C40—H40	107.7	C75—C76—C77	119.0 (8)
C40—C41—H41A	109.5	C75—C76—H76	120.5
C40—C41—H41B	109.5	C77—C76—H76	120.5
H41A—C41—H41B	109.5	C76—C77—C78	121.7 (7)
C40—C41—H41C	109.5	C76—C77—H77	119.2
H41A—C41—H41C	109.5	C78—C77—H77	119.2
H41B—C41—H41C	109.5	C73—C78—C77	118.5 (7)
C40—C42—H42A	109.5	C73—C78—H78	120.8
C40—C42—H42B	109.5	C77—C78—H78	120.8
H42A—C42—H42B	109.5	C73—C79—H79A	109.5
C40—C42—H42C	109.5	C73—C79—H79B	109.5
H42A—C42—H42C	109.5	H79A—C79—H79B	109.5
H42B—C42—H42C	109.5	C73—C79—H79C	109.5
C48—C43—C44	120.1 (3)	H79A—C79—H79C	109.5
C48—C43—N2	119.9 (3)	H79B—C79—H79C	109.5
			
C13—N1—C1—C11	2.0 (6)	C32—C33—C34—C35	1.0 (6)
C13—N1—C1—C2	179.6 (3)	C33—C34—C35—C36	0.5 (6)
C43—N2—C2—C3	−5.5 (6)	C34—C35—C36—C31	−1.1 (5)
C43—N2—C2—C1	−178.9 (3)	C34—C35—C36—C40	177.9 (4)
N1—C1—C2—N2	−4.0 (5)	C32—C31—C36—C35	0.3 (5)
C11—C1—C2—N2	174.1 (3)	C17—C31—C36—C35	−179.2 (3)
N1—C1—C2—C3	−178.9 (3)	C32—C31—C36—C40	−178.7 (3)
C11—C1—C2—C3	−0.8 (3)	C17—C31—C36—C40	1.8 (5)
N2—C2—C3—C4	2.9 (7)	C33—C32—C37—C39	−56.0 (5)
C1—C2—C3—C4	176.9 (4)	C31—C32—C37—C39	123.5 (4)
N2—C2—C3—C12	−172.4 (4)	C33—C32—C37—C38	69.3 (5)
C1—C2—C3—C12	1.6 (4)	C31—C32—C37—C38	−111.1 (4)
C12—C3—C4—C5	1.8 (6)	C35—C36—C40—C42	63.6 (5)
C2—C3—C4—C5	−173.2 (4)	C31—C36—C40—C42	−117.5 (4)
C3—C4—C5—C6	−2.3 (8)	C35—C36—C40—C41	−60.3 (5)
C4—C5—C6—C7	1.4 (10)	C31—C36—C40—C41	118.6 (4)
C5—C6—C7—C12	0.0 (8)	C2—N2—C43—C48	−84.7 (4)
C5—C6—C7—C8	178.7 (6)	C2—N2—C43—C44	96.4 (4)
C12—C7—C8—C9	0.8 (8)	C48—C43—C44—C45	2.1 (6)
C6—C7—C8—C9	−177.8 (6)	N2—C43—C44—C45	−179.1 (3)
C7—C8—C9—C10	−0.2 (9)	C43—C44—C45—C46	−1.1 (6)
C8—C9—C10—C11	0.5 (8)	C43—C44—C45—C49	179.6 (3)
C9—C10—C11—C12	−1.4 (6)	C44—C45—C46—C47	−0.6 (5)
C9—C10—C11—C1	176.4 (4)	C49—C45—C46—C47	178.7 (3)
N1—C1—C11—C10	−0.5 (7)	C45—C46—C47—C48	1.3 (5)
C2—C1—C11—C10	−178.3 (4)	C45—C46—C47—C61	−178.1 (3)
N1—C1—C11—C12	177.5 (4)	C46—C47—C48—C43	−0.3 (6)
C2—C1—C11—C12	−0.3 (4)	C61—C47—C48—C43	179.1 (3)
C6—C7—C12—C3	−0.4 (7)	C44—C43—C48—C47	−1.4 (6)
C8—C7—C12—C3	−179.3 (5)	N2—C43—C48—C47	179.8 (3)
C6—C7—C12—C11	177.0 (5)	C44—C45—C49—C54	102.4 (4)
C8—C7—C12—C11	−1.8 (7)	C46—C45—C49—C54	−76.9 (4)
C4—C3—C12—C7	−0.5 (6)	C44—C45—C49—C50	−79.4 (4)
C2—C3—C12—C7	175.7 (4)	C46—C45—C49—C50	101.3 (4)
C4—C3—C12—C11	−178.2 (4)	C54—C49—C50—C51	−1.2 (5)
C2—C3—C12—C11	−1.9 (4)	C45—C49—C50—C51	−179.4 (3)
C10—C11—C12—C7	2.1 (6)	C54—C49—C50—C55	177.5 (3)
C1—C11—C12—C7	−176.3 (4)	C45—C49—C50—C55	−0.7 (5)
C10—C11—C12—C3	179.8 (4)	C49—C50—C51—C52	1.0 (5)
C1—C11—C12—C3	1.4 (5)	C55—C50—C51—C52	−177.7 (4)
C1—N1—C13—C18	90.4 (4)	C50—C51—C52—C53	0.1 (6)
C1—N1—C13—C14	−93.0 (4)	C51—C52—C53—C54	−1.1 (6)
C18—C13—C14—C15	−1.1 (6)	C52—C53—C54—C49	0.9 (5)
N1—C13—C14—C15	−177.6 (3)	C52—C53—C54—C58	−177.8 (3)
C13—C14—C15—C16	0.3 (5)	C50—C49—C54—C53	0.3 (5)
C13—C14—C15—C19	−176.8 (3)	C45—C49—C54—C53	178.5 (3)
C14—C15—C16—C17	0.6 (5)	C50—C49—C54—C58	178.9 (3)
C19—C15—C16—C17	177.7 (3)	C45—C49—C54—C58	−2.9 (5)
C15—C16—C17—C18	−0.8 (5)	C51—C50—C55—C56	80.0 (8)
C15—C16—C17—C31	−179.2 (3)	C49—C50—C55—C56	−98.6 (8)
C14—C13—C18—C17	0.9 (6)	C51—C50—C55—C57	−70.2 (9)
N1—C13—C18—C17	177.4 (3)	C49—C50—C55—C57	111.2 (9)
C16—C17—C18—C13	0.1 (5)	C53—C54—C58—C60	−71.5 (5)
C31—C17—C18—C13	178.4 (3)	C49—C54—C58—C60	109.9 (4)
C16—C15—C19—C20	−99.2 (4)	C53—C54—C58—C59	52.8 (5)
C14—C15—C19—C20	77.8 (4)	C49—C54—C58—C59	−125.8 (4)
C16—C15—C19—C24	81.6 (4)	C48—C47—C61—C62	100.9 (4)
C14—C15—C19—C24	−101.4 (4)	C46—C47—C61—C62	−79.6 (5)
C24—C19—C20—C21	2.6 (5)	C48—C47—C61—C66	−79.7 (4)
C15—C19—C20—C21	−176.6 (3)	C46—C47—C61—C66	99.7 (4)
C24—C19—C20—C25	−177.3 (3)	C66—C61—C62—C63	−0.8 (6)
C15—C19—C20—C25	3.5 (5)	C47—C61—C62—C63	178.5 (4)
C19—C20—C21—C22	−1.1 (6)	C66—C61—C62—C67	179.2 (3)
C25—C20—C21—C22	178.8 (4)	C47—C61—C62—C67	−1.5 (5)
C20—C21—C22—C23	−0.8 (7)	C61—C62—C63—C64	0.6 (6)
C21—C22—C23—C24	1.3 (7)	C67—C62—C63—C64	−179.4 (4)
C22—C23—C24—C19	0.2 (6)	C62—C63—C64—C65	0.1 (7)
C22—C23—C24—C28	179.4 (4)	C63—C64—C65—C66	−0.7 (7)
C20—C19—C24—C23	−2.1 (5)	C64—C65—C66—C61	0.5 (6)
C15—C19—C24—C23	177.0 (3)	C64—C65—C66—C70	−176.7 (4)
C20—C19—C24—C28	178.7 (3)	C62—C61—C66—C65	0.3 (5)
C15—C19—C24—C28	−2.2 (5)	C47—C61—C66—C65	−179.0 (3)
C21—C20—C25—C26	61.0 (5)	C62—C61—C66—C70	177.4 (3)
C19—C20—C25—C26	−119.0 (4)	C47—C61—C66—C70	−2.0 (5)
C21—C20—C25—C27	−64.1 (5)	C63—C62—C67—C68	73.9 (12)
C19—C20—C25—C27	115.9 (4)	C61—C62—C67—C68	−106.1 (12)
C23—C24—C28—C30	46.7 (7)	C63—C62—C67—C69	−50.0 (9)
C19—C24—C28—C30	−134.1 (6)	C61—C62—C67—C69	129.9 (9)
C23—C24—C28—C29	−47.3 (6)	C65—C66—C70—C71	54.4 (5)
C19—C24—C28—C29	131.9 (5)	C61—C66—C70—C71	−122.6 (4)
C16—C17—C31—C32	76.7 (4)	C65—C66—C70—C72	−71.1 (5)
C18—C17—C31—C32	−101.7 (4)	C61—C66—C70—C72	111.9 (4)
C16—C17—C31—C36	−103.8 (4)	C78—C73—C74—C75	−0.9 (10)
C18—C17—C31—C36	77.9 (4)	C79—C73—C74—C75	178.0 (6)
C36—C31—C32—C33	1.1 (5)	C73—C74—C75—C76	0.7 (10)
C17—C31—C32—C33	−179.4 (3)	C74—C75—C76—C77	0.2 (11)
C36—C31—C32—C37	−178.5 (3)	C75—C76—C77—C78	−0.9 (15)
C17—C31—C32—C37	1.0 (5)	C74—C73—C78—C77	0.2 (13)
C31—C32—C33—C34	−1.7 (6)	C79—C73—C78—C77	−178.7 (8)
C37—C32—C33—C34	177.9 (3)	C76—C77—C78—C73	0.7 (15)
